# Transcriptional and biochemical analyses of gibberellin expression and content in germinated barley grain

**DOI:** 10.1093/jxb/erz546

**Published:** 2019-12-10

**Authors:** Natalie S Betts, Christoph Dockter, Oliver Berkowitz, Helen M Collins, Michelle Hooi, Qiongxian Lu, Rachel A Burton, Vincent Bulone, Birgitte Skadhauge, James Whelan, Geoffrey B Fincher

**Affiliations:** 1 Australian Research Council Centre of Excellence in Plant Cell Walls, School of Agriculture, Food and Wine, University of Adelaide, Waite Campus, Glen Osmond, SA, Australia; 2 Carlsberg Research Laboratory, Copenhagen V, Denmark; 3 School of Life Science and ARC Centre of Excellence in Plant Energy Biology, La Trobe University, Bundoora, Melbourne, VIC, Australia; 4 Adelaide Glycomics, School of Agriculture, Food and Wine, University of Adelaide, Glen Osmond, SA, Australia; 5 Royal Holloway, University of London, UK

**Keywords:** Aleurone, germination, gibberellic acid, *Hordeum vulgare*, RNA-seq, scutellum, signal transduction

## Abstract

Mobilization of reserves in germinated cereal grains is critical for early seedling vigour, global crop productivity, and hence food security. Gibberellins (GAs) are central to this process. We have developed a spatio-temporal model that describes the multifaceted mechanisms of GA regulation in germinated barley grain. The model was generated using RNA sequencing transcript data from tissues dissected from intact, germinated grain, which closely match measurements of GA hormones and their metabolites in those tissues. The data show that successful grain germination is underpinned by high concentrations of GA precursors in ungerminated grain, the use of independent metabolic pathways for the synthesis of several bioactive GAs during germination, and a capacity to abort bioactive GA biosynthesis. The most abundant bioactive form is GA_1_, which is synthesized in the scutellum as a glycosyl conjugate that diffuses to the aleurone, where it stimulates *de novo* synthesis of a GA_3_ conjugate and GA_4_. Synthesis of bioactive GAs in the aleurone provides a mechanism that ensures the hormonal signal is relayed from the scutellum to the distal tip of the grain. The transcript data set of 33 421 genes used to define GA metabolism is available as a resource to analyse other physiological processes in germinated grain.

## Teaser

Transcript and hormone measurements from germinated barley grain provide a model describing the complex signal transduction pathways of gibberellic acid.

## Introduction

The germination of barley (*Hordeum vulgare*) and other commercially important cereal and grass grains involves a complex set of interactions between the living aleurone and embryo tissues. The latter includes the scutellum and its associated scutellar epithelium layer, which lies at the interface of the embryo and the non-living starchy endosperm. Following the release of dormancy, germination of the grain is initiated by the uptake of water, mostly through the micropylar region of the embryo (Bewley and Black, 1994). A member of the gibberellin (GA) group of phytohormones is released from the embryo and diffuses from the proximal to the distal end of the endosperm (Yamaguchi, 2008). The scutellar epithelium appears to be the first GA target tissue, based on the detection there of α-amylase and (1,3;1,4)-β-glucanase gene transcripts and α-amylase enzyme activity (Gibbons, 1981; McFadden *et al.*, 1988). As the GA diffuses along the grain, it induces gene expression in the living aleurone cells of the endosperm, which form a thin layer around the non-living starchy endosperm (Duffus and Cochrane, 1993; Aubert *et al.*, 2018).

The germination process has attracted a high level of research interest because of its crucial importance in the establishment and vigour of the young seedling and hence in the ultimate yields and quality of cereal crops, which provide the major source of daily carbohydrate intake for many human societies. In addition, germinated cereal grains, in particular barley, have been used as the primary raw material for brewing beer and other alcoholic beverages for thousands of years.

Aleurone function is focused on the *de novo* synthesis of numerous hydrolytic enzymes, which depolymerize the cell walls, starch, storage proteins, and residual nucleic acids of the starchy endosperm (Fincher, 1989). The low molecular weight degradation products from the starchy endosperm are actively transported via the scutellum to the main body of the embryo, where they support the growth of the young seedling. Thus, the scutellar epithelium layer switches from secreting hydrolytic enzymes initially to become a major transporter of starchy endosperm degradation products into the embryo (Fincher, 2010).

Isolated aleurone layers from barley grain have been used extensively as a model system to study the effects of exogenous GAs and their antagonist abscisic acid (ABA) during grain germination. The hulless barley variety, Himalaya, has been particularly popular because viable aleurone layers are easier to dissect compared with those from hulled varieties. The isolated layers have been used to study the effects of exogenous GA_3_ both on hydrolytic enzyme secretion and on gene transcription (Chrispeels and Varner, 1967; Jacobsen and Varner, 1967; Lanahan *et al.*, 1992; Gubler *et al.*, 1995; Gómez-Cadenas *et al.*, 2001; Chen and An, 2006; An and Lin, 2011; Shahpiri *et al.*, 2015; Daneri-Castro *et al.*, 2016*a*, *b*).

While the data generated in these experiments provided new insights into the functions of GA, results from this isolated tissue approach could not be confidently extrapolated back to the much more complex series of interactions that occurs within and between the individual tissues of the whole, intact grain. Here, we have used a recently developed procedure to isolate tissues, in highly purified form, from intact barley grains germinated for up to 4 d, and this has allowed us to circumvent the interpretative problems associated with the use of isolated tissue preparations (Betts *et al.*, 2017a). We have defined transcript profiles in these tissues by RNA sequencing (RNA-seq) and validated the results by correlating the abundance of gene transcripts encoding enzymes of the GA metabolic pathways with the actual amounts of active GA species, GA conjugates, and intermediary metabolites in the tissues. In this way, we are able to define the sites of GA synthesis and the metabolic pathways through which the various forms of active GA are synthesized and degraded in the germinated grain. The combined transcript and hormone data have enabled the spatial and temporal complexities of GA metabolism in germinated grain to be described in a biological model at a level of detail not previously possible.

## Materials and methods

### Plant material

For RNA-seq analyses, *Hordeum vulgare* (cv. Navigator) grains (100) were germinated in the dark for 24, 48, 72, or 96 h at 16 °C as described by Betts *et al.* (2017a). Note that as a result of Green Revolution breeding advances, loss-of-function alleles such as the semi-dwarfing allele *sdw1.d* of the GA biosynthetic gene *GA20ox2* are widespread in modern spring barley germplasm (Xu *et al.* 2017). Sequencing the respective *GA20ox2* gene fragment in cv. Navigator (as described in Mascher *et al.*, 2017, using PCR primers 5'-GGTGCTCCAGACCGCTCAGC-3' and 5'-CCTCCGGAGGTCGTACACC-3') confirmed that the *GA20ox2* loss-of-function allele *sdw1.d* is present in cv. Navigator. As a result of the knock down of *GA20ox2*, the expression patterns of *GA20ox1* and *GA20ox3* change. In particular, some compensation for the loss of the *GA20ox2* gene is seen in the stem, where *GA20ox3* is highly expressed in these Green Revolution lines (Xu *et al.*, 2017) and transcript profiles might not necessarily match those observed in other barley varieties, including cv. Morex, which has an expressed *GA20ox2* gene (Mascher *et al.*, 2017).

For each time point, 10 grains were cut, fixed, and dissected as described previously (Betts *et al.*, 2017a), except that incubation in RNAlater™ (Sigma-Aldrich, USA) was found to be unnecessary and was omitted. Isolated tissues included the aleurone layer, the scutellum, and the remaining embryo (without the scutellum). Non-living tissues such as the starchy endosperm, the crushed cell layer, the pericarp–testa, and the husk contained very low levels of residual RNA (Betts *et al.*, 2017a) and were not examined here. Isolated aleurone layers were dissected into three approximately equal sections that included the proximal region (designated ‘al1’) from the embryo end of the grain, the central section (‘al2’), and the distal (‘al3’) section (Betts *et al.*, 2017a).

For hormone analyses, roots were removed from germinated grain for time points later than 24 h, and the whole embryo, including the scutellum, was collected separately from the rest of the grain for all time points. Two technical replicates were performed for each tissue and time point, except 96 h aleurone, where only one replicate was available. Biological variation was captured within samples, each of which comprised 20–150 grains.

### RNA sequencing analyses

RNA was prepared and sequenced from three replicate samples, each comprising material from 10 grains, to cover biological variation. RNA was isolated from 30–200 mg of the living tissues, using the Spectrum™ Plant Total RNA kit (Sigma-Aldrich) (Betts *et al.*, 2017a). RNA integrity was assessed using an Agilent 2200 TapeStation system (Agilent Technologies, Inc., Germany). Libraries and RNA-seq analyses were performed as described by Betts *et al.* (2017a), except that 60 bp single reads were used, and genes in both high and low confidence categories were analysed. For analysis of functional gene categories, the PageMan tool was used (Usadel *et al.*, 2006) after gene annotation with the Mercator pipeline (Lohse *et al.*, 2014) using reference plant genomes and subsequent manual curation.

Transcript abundances and count estimates [transcripts per million (TPM)] were determined using a k-mer index build from the representative transcript models (Mascher *et al.*, 2017) using a k-mer length of 31 and the kallisto program (version 0.46.0) with 100 bootstraps (Bray *et al.*, 2016). Only genes with at least five counts in a quarter of all samples were further analysed. A likelihood ratio test was used to test for differential gene expression using the sleuth software (version 0.29.0) (Pimentel *et al.*, 2017). Differentially expressed genes (DEGs) were called with a false discovery rate (FDR) <0.05 and a log_2_ fold change (FC) >1. Overlaps in the list of DEGs across the different genotypes were identified and represented using UpSetR (version1.4.0) (Conway *et al.*, 2017). For further analyses, hierarchical clustering and generation of heat maps with the Partek Genomics software suite version 6.16 (Partek Incorporated, http://www.partek.com/) was used. Gene Ontology (GO) enrichment analysis of the gene lists (downloaded from the IPK/IBSC website https://webblast.ipk-gatersleben.de/barley_ibsc/) was carried out the using ‘BiNGO’ plugin for Cytoscape (version 3.0.3) (Maere *et al.*, 2005) and considered statistically significant with a *P*<0.05 after Bonferroni correction.

### *In silico* gene discovery

Genes known to be involved in GA metabolism were initially identified through the literature (Hedden and Kamiya, 1997; Spielmeyer *et al.*, 2004; Magome *et al.*, 2013; Pearce *et al.*, 2015). Candidate genes in barley were subsequently identified via annotations in the barley genome (Mascher *et al.*, 2017) and in GenBank (https://www.ncbi.nlm.nih.gov/genbank/). Further candidates were found by nucleotide and protein homology searches on the BLAST server (high and low confidence genes) of the Leibniz Institute of Plant Genetics and Crop Plant Research (IPK), Gatersleben, Germany (http://webblast.ipk-gatersleben.de/barley_ibsc/).

### Microscopy

Tissue samples were fixed, sectioned, and either stained or labelled for light microscopy and photographed using a Carl Zeiss M2 AxioImager microscope (Betts *et al.*, 2017b; Aubert *et al.*, 2018). Sections stained with toluidine blue O (Sigma-Aldrich) were photographed using digital interference contrast (DIC). Calcofluor white- (Sigma-Aldrich) stained sections were viewed using Zeiss Filter set 49 (excitation 335–383 nm, emission 420–470 nm, exposure 230 ms; false-coloured turquoise). Immunofluorescence labelling of (1,3;1,4)-β-glucan and arabinoxylan was performed with the BG1 antibody (Meikle *et al.*, 1994) and the LM11 antibody (McCartney *et al.*, 2005), respectively, as described by Burton *et al.* (2011), with the minor modifications outlined in Betts *et al.* (2017b). Fluorescence labelling was observed using a Zeiss Fluorescence microscope (Axio Imager M2, Zeiss, Germany) with an AxioCam Mrm camera and processed using ZEN 2012 software (Zeiss, Australia).

### Hormone quantification in grain samples

Frozen, ground plant tissue (750±100 mg) was extracted with LC/MS-grade methanol (Fisher Chemical, USA) at a sample:solvent ratio of 1:10 (mg µl^–1^); that is, 750 mg of powdered tissue in 7.5 ml. To each sample was added 300 ng of deuterated GA_1_ internal standard (Olchemim, Czech Republic). After shaking at 0 °C for 30 min, 12 ml of HPLC-grade dichloromethane (Sigma-Aldrich) was added and the mixtures were gently shaken at 0 °C for 45 min, and centrifuged at 1000 *g* for 30 min at 4 °C before drying the supernatant under nitrogen. Samples were re-dissolved in 600 µl of methanol and incubated at 4 °C for 30 min before centrifuging at 16 000 *g* for 30 min at 4 °C. The supernatant was transferred to a 1.5 ml tube, dried under nitrogen, and re-dissolved in 10 μl of methanol.

This solution (2.5 µl) was injected into an ultra-performance liquid chromatography (UPLC) system coupled to a 6545 quadrupole time-of-flight (QTOF) mass spectrometer (Agilent Technologies, Singapore). A C18 guard column (1.8 μm particle size, 2.1 mm inner diameter, 10 mm long) was connected to the analytical C18 reverse phase column (1.8 μm particle size, 2.1 mm inner diameter, 100 mm long; Agilent Technologies, USA). The samples were separated at a flow rate of 300 μl min^–1^ using water:formic acid (99.9:0.1, v/v) as solvent A and methanol:formic acid (99.9:0.1, v/v) as solvent B in a stepwise gradient at 20 °C: 2–48% B (0–30 min), 48–90% B (30–70 min), held at 90% B for 5 min, and finally at 2% B for 15 min.

The UPLC system was connected to the QTOF mass spectrometer via a dual Agilent Jetstream electrospray interface (Dual AJS ESI) and analyses were conducted in both positive and negative modes. A capillary voltage of 4 kV was applied. The gas temperature used at the source was 325 °C, with a flow rate of 8 l min^–1^. The sheath gas temperature was 400 °C, with a flow rate of 10 l min^–1^. Data-dependent acquisitions of mass spectra with a full scan (*m/z* 50–1100) were accomplished in centroid mode. Purine and hexakis were used as reference lock-mass compounds with automatic mass correction enabled, using the Agilent Mass Hunter Qualitative software (B.01.03).

Phytohormones were identified using the Agilent Technologies MassHunter Workstation software (B.08.00). Gibberellins GA_1_, GA_3_, GA_4_, GA_7_, GA_20_, GA_51_, and GA_53_, and *ent*-kaurenoic acid standards (Olchemim) provided retention time and adduct information. For all GA molecules, identification was confirmed manually using characteristic *m/z* transitions identified in previous multiple reaction monitoring (MRM) studies (Supplementary Table S1 at *JXB* online; Chiwocha *et al.*, 2003; Urbanová *et al.*, 2013; Delatorre *et al.*, 2017). We used an approach based on peak areas relative to a consistent internal standard to normalize recovery and response rates of different GA-related analytes throughout the extraction and detection processes. Variations over time of GA glycoconjugates, *ent*-kaurene, and *ent*-kaurenoic acid were estimated based on peak area ratios in the UPLC chromatograms. Mass quantification of GAs was based on peak area comparisons with the deuterated GA_1_ internal standard (300 ng) within each individual sample, and converted to molar amounts based on the molecular weights for each GA species.

## Results

### Morphological changes show the different fates of the aleurone and scutellum epithelium

Germination has been defined as the period between initiation of the process, achieved through wetting the grain, to the point of coleorhiza emergence (Bewley and Black, 1994). The latter occurred in our grain samples at 1–2 d and we have therefore used the term ‘germinated’, rather than ‘germinating’, throughout this 0–4 d study.

**Fig. 1.**
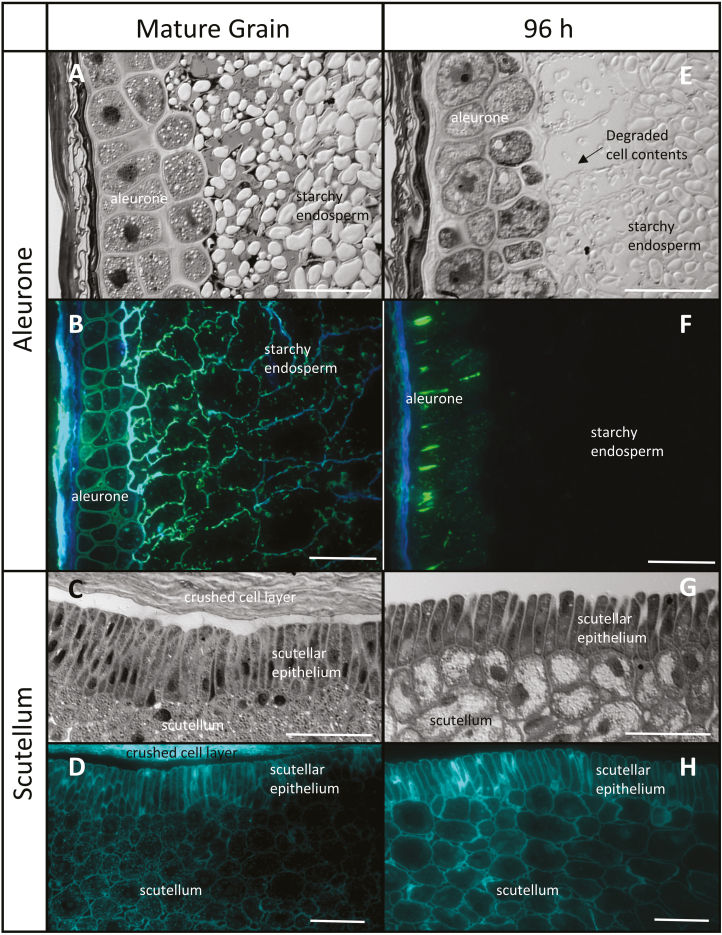
Comparative morphology of the aleurone layer and scutellum of mature and germinated barley grain. (A and E) Aleurone sections stained with toluidine blue and photographed using differential interference contrast (DIC) microscopy, showing the degradation of the subaleurone starchy endosperm between 0 h and 96 h. (B and F) Immunocytochemistry of aleurone arabinoxylan and (1,3;1,4)-β-glucans using LM11 (blue) and BG1 (green) antibodies. (C and G) Scutellum sections stained with toluidine blue and photographed using DIC microscopy, showing the lateral separation of the epithelial cells after 96 h. (D and H) Calcofluor staining of scutellum (turquoise). Scale bar=50 μm.

In the ungerminated barley grain, the aleurone layer, which is distinguished by its thick cell walls, is 2–3 cells in thickness and the granular nature of the aleurone cell contents is evident (Fig. 1A). The cells of the starchy endosperm are larger and their walls are thinner (Fig. 1B). At 96 h after imbibition, gaps have opened between the aleurone cells (Fig. 1E), extensive loss of the aleurone wall is evident (Fig. 1F), and the wall network in both the aleurone and starchy endosperm cells has largely disappeared (Fig. 1F). Residual wall material is observed in some anticlinal walls of the aleurone layer (Fig. 1F).

After 96 h, the columnar scutellar epithelial cells have disconnected from each other along their lateral cell walls (Fig. 1C, G), but the overall cell wall network of the embryo appears similar to that in the mature grain (Fig. 1D, H).

### Transcript data identify key members of gene families transcribed in the aleurone and the scutellum

High quality RNA (average integrity numbers >7) was isolated at 0, 24, 36, 72, and 96 h from the scutellum, the residual embryo, and three aleurone sections (the proximal al1 section, the central al2 section, and the distal al3 section; Betts *et al.*, 2017a). The complete data set of transcripts from 33 421 genes, expressed as TPM, is presented for all genes, tissues, and time points in Supplementary Table S2A. In addition, transcripts of genes specifically mentioned here are listed in Supplementary Table S3.

Transcript data for the aleurone were consistent with its primary role in the synthesis and secretion of enzymes that mediate the mobilization of the starchy endosperm. The al1 data set showed abundant transcripts at 24 h for genes encoding hydrolytic enzymes such as carboxypeptidases (HORVU6Hr1G006880, 7066 TPM; HORVU7Hr1G059850, 2075 TPM), cysteine proteinases (HORVU3Hr1G091800, 2975 TPM; HORVU4Hr1G010390, 2273 TPM; HORVU3Hr1G091920, 1130 TPM), (1,3;1,4)-β- glucanase isoenzyme EI (HORVU1Hr1G057680, 1649 TPM), arabinoxylan arabinohydrolase (HORVU5Hr1G045150, 1617 TPM), β-glucan exohydrolase (HORVU5Hr1G095130, 1362 TPM), and α-amylase (HORVU6Hr1G080790, 556 TPM). Transcripts of the (1,3)-β-glucanase genes HORVU3Hr1G105600 and HORVU3Hr1G105630 are also abundant (up to 581 TPM and 783 TPM by 96 h, respectively; Supplementary Table S3).

The transcript data from the scutellum reflected its dual secretory and transport functions. Most of the transcripts in the scutellum data set at 0 h encoded metabolic enzymes. However, from 24 h onwards, transcripts for hydrolytic enzymes became much more abundant and included transcripts for cysteine protease genes (HORVU3Hr1G091800*; HORVU3Hr1G091920*; HORVU2Hr1G113460; HORVU2 Hr1G121440; and HORVU4Hr1G010390*), a carboxypep- tidase gene (HORVU3Hr1G096830), an α-amylase gene (HORVU6Hr1G080790*), and a (1,3;1,4)-β-glucanase gene (HORVU1Hr1G057680*) (Supplementary Table S3). The asterisks indicate that these gene transcripts were the same as those that were highly abundant in the aleurone layer.

### A wave of expression occurs from the proximal to the distal aleurone

Many of the transcripts that increased in abundance in the aleurone layer, including those listed above, have transcript patterns that peaked ~24 h later in the aleurone al2 tissue extracts than in the proximal aleurone al1 tissues and later still in the distal aleurone al3 tissues (Supplementary Table S2A).

**Fig. 2.**
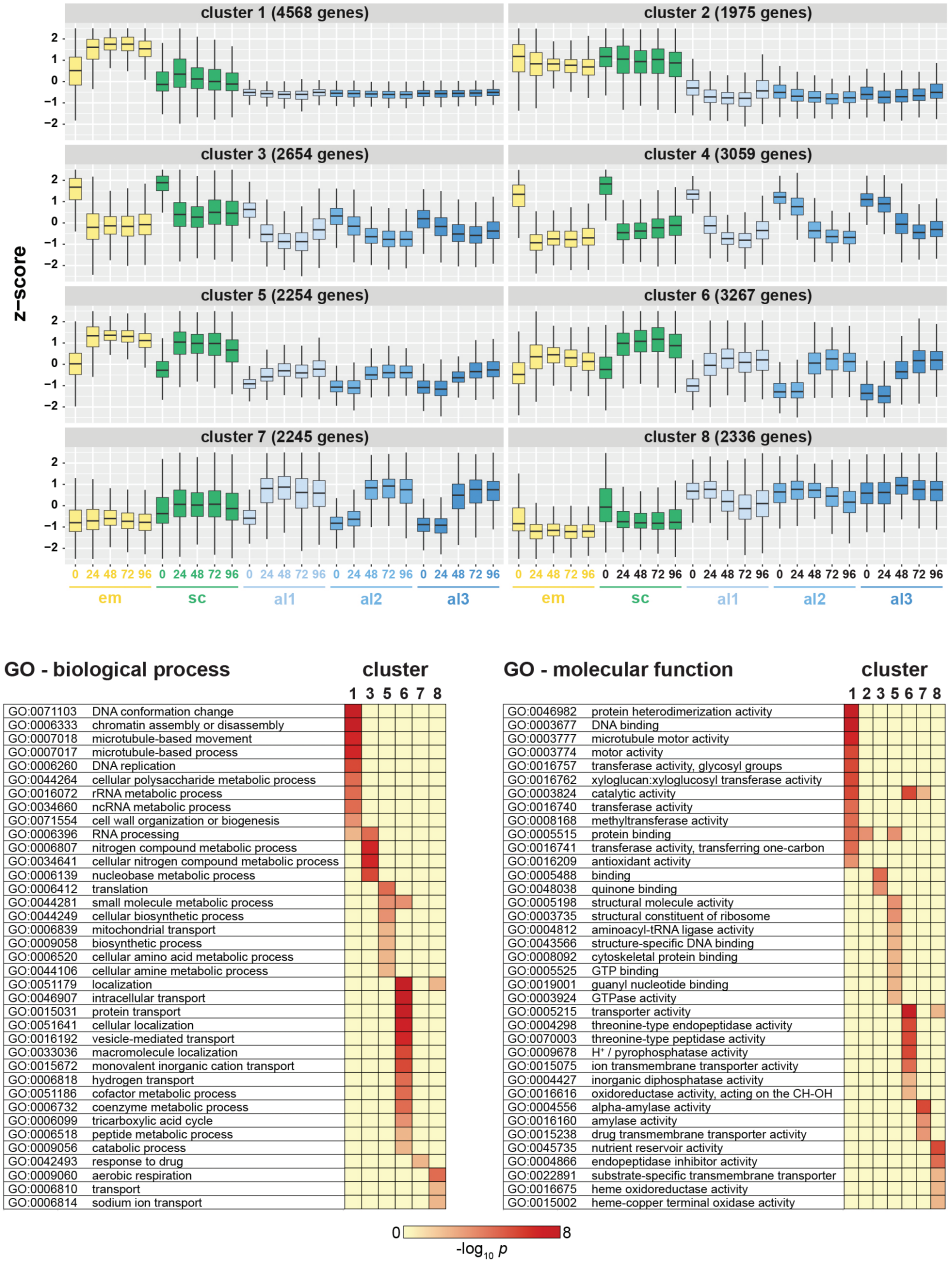
Co-expression of barley genes across tissues and time points. (A) Gene clustering using a self-organizing maps algorithm with 10 000 training iterations identified eight clusters of differentially expressed genes with similar expression patterns in the analysed tissue types and time points. The number of clusters was optimized for limited redundancy in co-expression patterns across clusters. Expression values in TPM were converted into z-scores before clustering. Numbers of genes in each cluster are as indicated. (B) For genes in each cluster, a GO term enrichment analysis (*P*<0.05 after Bonferroni correction) was performed for the ‘biological process’ and ‘molecular function’ ontologies. Scale indicates the –log_10_*P*-value for GO term enrichment.

A global cluster analysis of transcripts expressed in all tissues at the five time points revealed eight co-expressed groups with different transcript abundance patterns (Fig. 2A). The cluster analyses confirmed the wave of gene activation from the proximal to the distal end of the aleurone, where the lag phases between the aleurone al1, al2, and al3 tissues are clearly apparent in all clusters, except clusters 1 and 8 where genes are constitutively expressed at low and high levels, respectively (Fig. 2A). The lag phase is evident both in genes that increase in expression levels during germination (clusters 5, 6, and 7) and in those that decrease in abundance over the 96 h period (clusters 3 and 4). Similarly, principal component analysis indicated major developmental changes within the first 24 h, where the aleurone al1, al2, and al3 tissues were seen in an extended cluster and show a progressive sequence along the second dimension from 0 h to 96 h (Supplementary Fig. S1).

Subsequent GO enrichment analysis of the clusters indicated that temporal changes of gene transcript abundances in clusters 6 and 7 reflect the secretory function of both the aleurone and the scutellum, where GO terms in the ‘molecular function’ category representative of α-amylase and endopeptidase activity are enriched (Fig. 2B). In addition, clusters 6 and 7 are enriched in localization and vesicle-mediated transporter protein genes, consistent with scutellum function. Genes of cluster 8, with consistently high expression across all aleurone sections, are enriched for GO terms ‘nutrient reservoir activity’, ‘heme oxidoreducatase’, and ‘aerobic respiration’, in agreement with the function of the aleurone in mobilizing its own reserves for enzyme biosynthesis.

### Differentially expressed genes indicate temporal coordination of gene expression

The highest numbers of DEGs were detected in the embryo, the scutellum, and the aleurone al1 tissues, with smaller numbers in al2 and al3 (Supplementary Fig. S2; Supplementary Table S2B). In the aleurone tissues, the number of DEGs increased over time (Supplementary Fig. S2). Overlaps (or intersects) of DEGs between the five grain tissues and between time points of aleurone al1 tissue (UpSet plots; Lex *et al.*, 2014) are shown in Supplementary Fig. S3A and B, respectively.

Across all tissues, 8102 DEGs were found to be common, with the embryo and scutellum sharing the highest number of common DEGs (Supplementary Table S2B; Supplementary Figs. S3A, B). Analysis of DEGs for the al1 tissue (Supplementary Table S2B) revealed that 25% of DEGs were up- or down-regulated at all time points after 0 h, indicating a large and ongoing shift in gene expression in the aleurone tissue. The vast majority of aleurone al1 DEGs were found at adjoining time points (e.g. 24 h and 48 h, 48 h and 72 h), confirming significant temporal coordination of gene expression in this tissue (Supplementary Table S2B).

### Spatial differences of expression occur in adjacent regions of the aleurone (al1) and scutellum

**Fig. 3.**
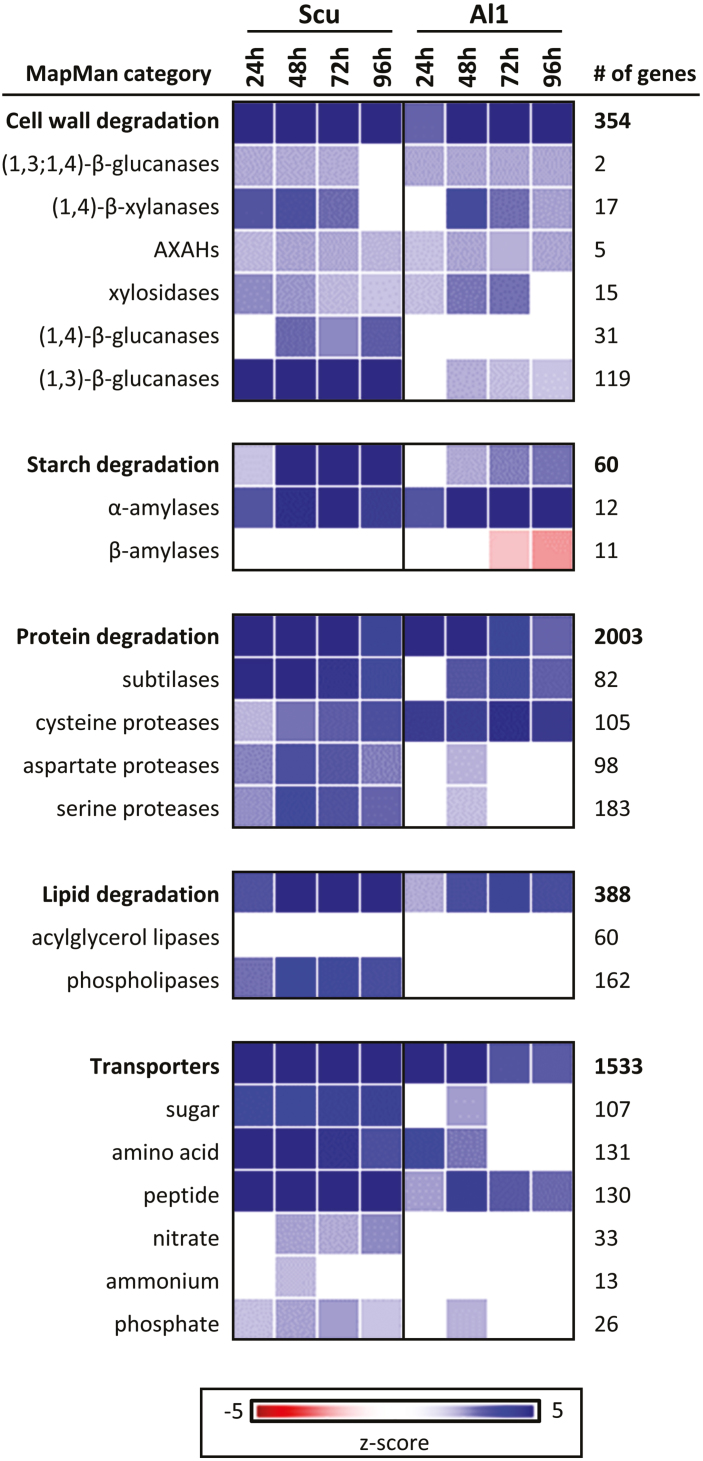
Gene expression in the aleurone al1 and scutellum. Using the PageMan tool, the difference in median fold change (relative to the 0 h time point) in gene expression for a particular category of genes was determined by applying a Wilcoxon rank sum test and converting resulting *P*-values to z-scores (Usadel *et al.*, 2006). For the aleurone (Al1) and scutellum (Scut) tissues, the major categories of encoded enzymes are shown. The top line in each panel gives the overall pattern for genes in that category, which is broken down into selected gene categories below. The number of genes represented in each category is given on the right.

Transcripts for cell wall-degrading enzymes were up-regulated in adjacent al1 and scutellum tissues, sometimes after an initial lag period of 1 d. For example, α-amylase gene transcripts increased to high levels in both tissues (Fig. 3). Transcript levels peaked in aleurone al1 at 72 h, with similar patterns detected in the scutellum, albeit at much lower levels (Supplementary Table S3). In addition, protease-encoding genes were up-regulated in both the aleurone al1 and scutellum at 24 h, with transcript levels of subtilisin-like proteases and cysteine proteases increasing in both tissues. One subtilisin-like protease, encoded by HORVU5Hr1G097150, exhibited very large transcript increases in the scutellum and aleurone al1 tissues, with peak transcript levels of >3000 TPM in aleurone al1 at 48 h (Supplementary Table S3). In contrast, aspartate protease and serine protease transcription increased predominantly in the scutellum, where transcripts of the HORVU6Hr1G084770 gene increased to a peak of 383 TPM at 48 h, while levels in aleurone al1 remained at <10 TPM. Transcripts of one serine carboxypeptidase gene (HORVU3Hr1G096830) were abundant in both the aleurone (up to 420 TPM) and the scutellum (up to 6060 TPM).

A noteworthy observation from the heat map in Fig. 3 is the up-regulation of sugar, amino acid, and peptide transporters in the scutellum, many of which were not detected in aleurone cells.

### Genes involved in gibberellin metabolism are identified

**Fig. 4.**
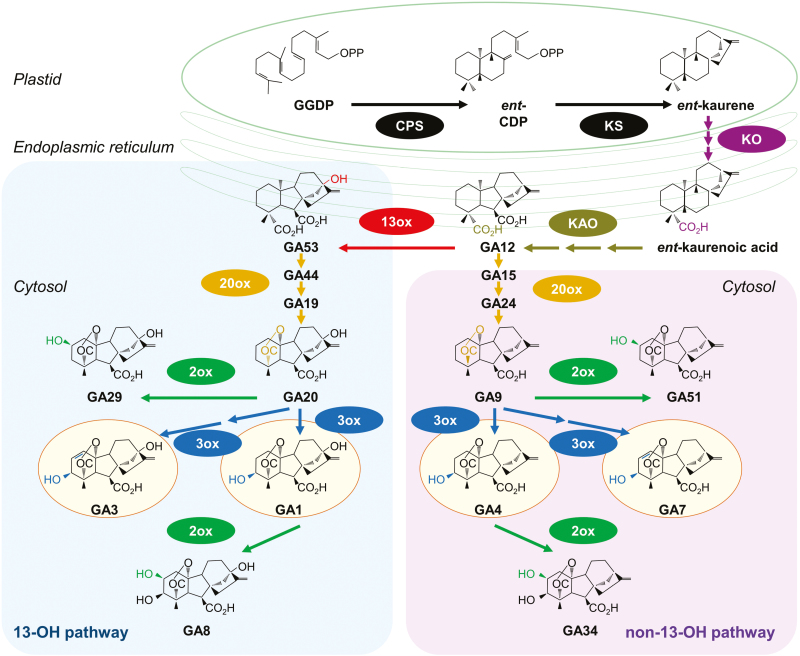
Metabolic pathways leading to active GA biosynthesis. Modified redrawn from Hedden and Thomas (2012) and Yamaguchi (2008). The active forms of GA are in yellow ovals; the GA oxidase enzymes are abbreviated as 13ox, 2ox, and 3ox. The enzymes *ent*-copalyl diphosphate synthase (CPS), *ent*-kaurene synthase (KS), *ent-*kaurene oxidase (KO), and *ent-*kaurenoic acid (KAO) are indicated, together with the metabolites geranylgeranyl diphosphate (GGDP) and *ent*-copalyl diphosphate (*ent-*CP). The primary breakdown products of the active GA_1_ and GA_4_ forms are also shown (GA_8_ and GA_34_). The coloured bonds and atoms in the chemical structures indicate the modifications catalysed by the enzyme shown in the same colour (Yamaguchi, 2008). Isoform numbers are not included, as they appear to be distributed spatially rather than segregating between the two pathways (Suzuki *et al.*, 2005). Note that some oxidized C-20 intermediates might not be released by the enzymes during some of the multistep reactions (Hedden and Sponsel, 2015). Recently, Liu *et al.* (2019) showed that a 2-oxoglutarate-dependent dioxygenase catalysed the formation of a previously unknown, biologically active gibberellin (DHGA_12_) in seeds of *Arabidopsis thaliana*, by hydration of GA_12_.

**Fig. 5.**
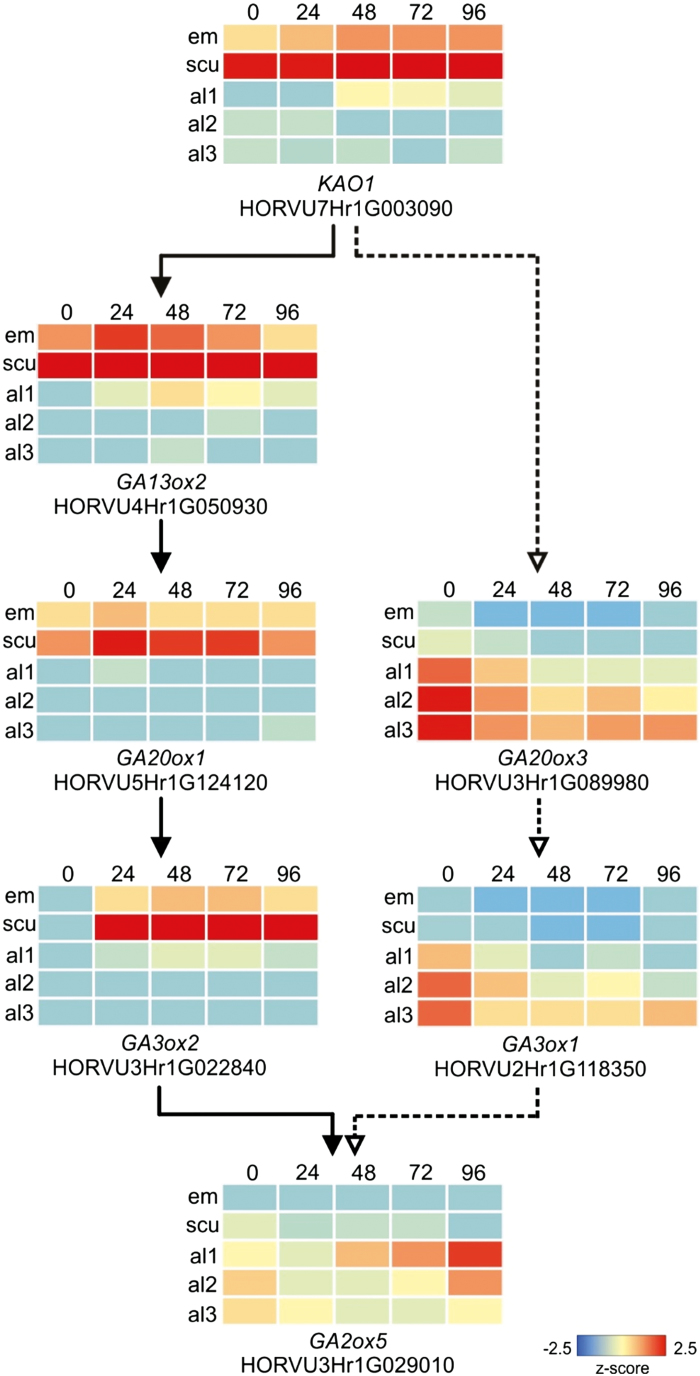
Expression levels of genes catalysing key steps of GA biosynthesis and catabolism. Embryo/scutellum (solid line path) or aleurone tissues (dotted line path) at 0, 24, 48, 72, and 96 h post-imbibition. Expression in transcripts per million (TPM) was converted to z-scores

Some 39 genes involved in GA metabolism were identified by nucleotide and protein homology searches, based on known gene sequences (Supplementary Table S4; Hedden and Kamiya, 1997; Spielmeyer *et al.*, 2004; Magome *et al.*, 2013; Pearce *et al.*, 2015; Mascher *et al.*, 2017). The biochemical pathways of active GA biosynthesis and degradation are summarized in Fig. 4, and heat maps of transcription patterns of key GA-related genes are shown in Fig. 5, where the differences between transcription in the aleurone and scutellum are clearly evident.

The most abundant GA-related transcript was *GA**13**ox2* (HORVU4Hr1G050930), which increased from 25 TPM in the scutellum at 0 h to 619 TPM at 48 h, before dropping back to 208 TPM at 96 h (Supplementary Table S3). This gene encodes the enzyme that converts GA_12_ to GA_53_ and commits GA biosynthesis to the 13-OH pathway, and hence to synthesize the active GA_1_ and/or GA_3_ forms of the hormone (Fig. 4). As expected, one of the *GA20ox* genes (HORVU5Hr1G124120), which encodes the enzyme that converts GA_53_ to GA_20_ along the 13-OH pathway, was also well represented in the scutellum.

The second most abundant transcript in the pathway encodes a GA3ox enzyme (HORVU3Hr1G022840), which converts the GA_20_ precursor to active GA_1_ or GA_3_ via the 13-OH pathway or precursor GA_9_ to active GA_4_ via the non-13-OH pathway (Fig. 4). These *GA3ox* transcripts increased in the scutellum, from 0 TPM at 0 h to 299 TPM at 72 h, and subsequently decreased to 174 TPM at 96 h (Supplementary Table S2A). Transcripts for the *GA20ox3* gene (HORVU3Hr1G089980), which encodes the enzyme that converts either GA_12_ to GA_9_ in the non-13-OH pathway towards bioactive GA_4_, or GA_53_ to GA_20_ in the 13-OH pathway towards bioactive GA_1_ and GA_3_ (Fig. 4), were detectable at low levels in aleurone cells, particularly in the al2 and al3 tissues, but not in the scutellum. Some of these transcripts might have originated from the mature grain (Pearce *et al.*, 2015; Mascher *et al.*, 2017).

The *KAO1* gene (HORVU7Hr1G003090) encodes the enzyme that oxidizes *ent*-kaurenoic acid to GA_12_ in the chloroplast envelope in the early steps of the pathway (Fig. 4; Helliwell *et al.*, 2001). Transcripts for this gene in the germinated grain were found at relatively high levels in the scutellum, with 20 TPM at 0 h and 268 TPM at 48 h, before decreasing to 89 TPM at 96 h (Supplementary Table S3).

Genes encoding central GA signalling components, such as the GA receptor protein GID1 (HORVU1Hr1G060810) and the DELLA protein (HORVU4Hr1G006930), were transcribed in both tissues, at levels ranging from 18 to 147 TPM and from 23 to 370 TPM, respectively. This is consistent with earlier observations that GA induces hydrolytic enzyme synthesis and secretion *in vitro* from both isolated aleurone layers and isolated scutella (Chrispeels and Varner, 1967; Stuart *et al.*, 1986). The early and steep increase of transcripts in the aleurone for the *DELLA* gene (45–370 TPM within 96 h), which is a key repressor in GA signalling and is involved in GA homeostasis, indicates very early GA signal transduction and regulation (Sun, 2011).

### Hormone analyses indicate high levels of stored precursor metabolites of gibberellins

When metabolites involved in the early steps of the diterpenoid biosynthetic pathway from geranylgeranyl diphosphate (GGDP) to active GAs were examined (Fig. 4; Supplementary Table S5), GGDP and *ent*-copalyl diphosphate (*ent*-CDP) could not be detected, but *ent*-kaurene and *ent*-kaurenoic acid were found in the embryo and aleurone layers (Supplementary Table S5). In the embryo, high levels of *ent*-kaurenoic acid at 0 h decreased to undetectable levels by 6 h; only very low levels of *ent*-kaurenoic acid were found in the aleurone. Levels of *ent*-kaurene were very high in the embryo and remained high for the 96 h germination period. *ent*-Kaurene levels were also detectable in significant amounts at 0 h in the aleurone and thereafter increased steadily to very high levels at 72 h (Supplementary Table S5).

Although the inactive, precursor GA intermediates GA_12_, GA_9_, and GA_20_ could not be detected at any stage in any tissue, the GA_53_ precursor of the 13-OH pathway (Fig. 4) was present in relatively high concentrations in the aleurone throughout the germination period; it also accumulated in the embryo after 6 h (Supplementary Table S5). In addition, the GA_19_ intermediate in the GA20ox conversion of GA_53_ to GA_20_ in the 13-OH pathway (Fig. 4; Yamaguchi, 2008) was detectable in the aleurone, but not the embryo (Supplementary Table S5). Barrero *et al.* (2013) also detected GA_19_ in the aleurone of germinated wheat grain and reported that its levels were greatly elevated in varieties showing the late maturity α-amylase phenotype.

**Fig. 6.**
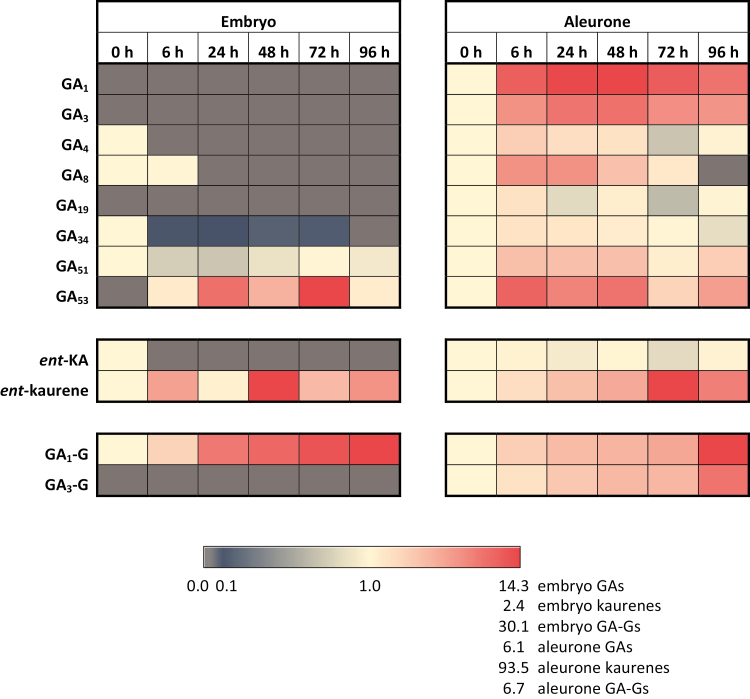
Heat maps showing changes in levels of GAs, GA-glycosides, and *ent*-kaurene/*ent*-kaurenoic acid in embryo and aleurone from 0 h to 96 h after the initiation of germination. Units are fold changes compared with the 0 h time point. Values <1 indicate reduced levels and those >1 indicate increased levels. A grey cell indicates a zero value.

In view of the difficulties in precisely quantitating very small amounts of GAs and related metabolites extracted with different efficiencies from different tissues, the values of the detected hormones and metabolites shown in Supplementary Table S5 must be considered semi-quantitative. However, in studies in Arabidopsis, it has been shown that recovery rates of a wide range of phytohormones ranged from 80% to 100% (Chiwocha *et al.*, 2003; Ross *et al.*, 2004; Šimura *et al*., 2018),

In the present study, we have defined when and where the various metabolites are found and have monitored changes relative to their 0 h time point values (Fig. 6). Because the *ent*-kaurene/*ent*-kaurenoic acid levels and progressive changes were much higher than those observed for the GAs and the GA-glycosides, the three groups are presented separately so that changes in each group could be observed more clearly (Fig. 6).

### GA_1_ in conjugated form is the most abundant gibberellin and is synthesized in the embryo

Of the four biologically active GA forms, GA_1_, GA_3_, and GA_4_ were detectable in the extracts, but the fourth active hormone, GA_7_, could not be detected in any of the tissues at any time (Supplementary Table S5). The most abundant GA component was a hexosyl conjugate of GA_1_, which increased from low levels in the embryo at 0 h to relatively high levels at 96 h; the GA_1_ conjugate also accumulated in the aleurone, albeit to a lesser extent (Fig. 6; Supplementary Table S5). The free forms of GA_1_ and GA_3_, together with a hexosyl conjugate of GA_3_ (GA_3_-G), were present in the aleurone throughout the 96 h period, but were not detectable in the embryo (Fig. 6; Supplementary Table S5).

The identities of the GA-glycosides were confirmed by MS, where their molecular ions of *m/z* 509 and *m/z* 507 corresponded to the molecular ions of non-glycosylated GA_1_ (*m/z* 347) and non-glycosylated GA_3_ (*m/z* 345), plus the anhydroglycose fragment of *m/z* 162 (Supplementary Fig. S4). In addition, several product ions that corresponded to characteristic *m/z* transitions for GA_1_ (*m/z* 259 and 303) and GA_3_ (*m/z* 221, 239, and 301) were also present in the GA-G spectra.

### Active turnover of bioactive gibberellins is occurring

The degradation products of active and precursor GAs were detectable in these analyses, namely GA_8_ from the 13-OH pathway and GA_34_ and GA_51_ from the non-13-OH pathway (Fig. 4). The GA_8_ degradation product was found mostly in the aleurone, while GA_34_ and GA_51_ were found in both the aleurone and embryo extracts (Supplementary Table S5). The occurrence of GA_8_ and GA_34_ indicates that active GA_1_ and GA_4_ are being degraded, respectively, while that of the GA_51_ indicates that the non-13-OH pathway can be aborted before the production of active GA_4_ (Fig. 4). Although the GA_29_ degradation product from GA_20_ could not be unequivocally identified, we cannot rule out the possibility that this GA_29_ degradation product might be present. The large amount of GA_4_ found in the embryo of ungerminated grain disappears within 6 h, which is consistent with the high levels of its degradation product GA_34_ in the scutellum and transcription of a *GA2ox* gene, HORVU1Hr1G086810, in the embryo (Supplementary Table S5).

## Discussion

A recently developed procedure that allows isolation and analysis of individual tissues from intact, germinated barley grain (Betts *et al.*, 2017a) was applied here to generate transcript profiles, over 4 d, of the living tissues of the germinated grain. The data matched the morphological changes and the known functions of the various tissues, as outlined above. The RNA-seq data also identified the most important individual members of gene families and their corresponding isoenzymes for the mobilization of cell walls, starch, and storage protein in the starchy endosperm. These results provide a ‘biological validation’ of the method, which was previously validated at the technical level by quantitative PCR analysis of selected genes (Betts *et al.*, 2017a).

However, the central objective of the work was to integrate these and previous data into a model that explained the complex but not well understood metabolism and signal transduction of GAs in the germinated grain. Although the GA antagonist, ABA, was detected in the grain (data not shown) and transcripts of genes involved in ABA metabolism were also present (Supplementary Table S2), this study was focused on the spatio-temporal regulation of GA biology in non-dormant grain under ideal germination conditions. Interactions of GA, ABA, and other metabolites in the context of abiotic stress tolerance and grain dormancy (Nambara and Marion-Poll, 2005; Dave *et al.* 2016; Urbanová and Leubner-Metzger, 2018) will be the subject of a future investigation.

The RNA-seq and hormone data closely match each other and have enabled us to address and answer several key, unresolved questions regarding GA function in germinated cereal grains, including: (i) the source and identities of precursor metabolites in the GA biosynthetic pathways; (ii) the precise sites of synthesis of the hormone in the grain; (iii) the biochemical pathways leading to the active forms; (iv) the active form(s) that cause the observed changes in gene expression; (v) whether or not turnover of active forms occurs; and (vi) whether or not the target aleurone layer itself produces any GA *de novo*, for transmission towards the distal end of the grain.

The first question relates to the source of diterpenoid precursors for GA biosynthesis, which are synthesized in the plastid envelope and the endoplasmic reticulum (Fig. 4; Aach *et al.*, 1997; Helliwell *et al.*, 2001; Hedden and Thomas, 2012). The grain was germinated in darkness, so functional chloroplasts would not be present. The hormone and metabolite analyses showed that high concentrations of the precursors *ent*-kaurene, *ent*-kaurenoic acid, and GA_53_ are present in the ungerminated grain (Supplementary Table S5). Murphy and Briggs (1973) also identified *ent*-kaurene in mature, dry grain of *Hordeum distichon* and showed that the amount decreased during germination. Here, *ent*-kaurenoic acid is found at high concentrations in the embryo at 0 h but disappears by 6 h. The presence of transcripts for kaurene oxidase (HORVU0Hr1G021760) and kaurenoic acid oxidase (HORVU7Hr1G003090) in both the scutellum and aleurone is consistent with the conversion of the stored precursors along pathways towards active GAs (Supplementary Table S2; Fig. 4). The final inactive precursor, GA_53_, which feeds into the 13-OH pathway, is present from 0 h in the aleurone and 6 h in the embryo, and rises to high levels in both tissues during the 96 h experiment (Supplementary Table S5).

The second question relates to the site of GA synthesis in the germinated barley grain, where the embryonic axis (MacLeod and Palmer, 1967), the scutellum (Radley, 1967; Yamaguchi, 2008), and the aleurone (Atzorn and Weiler, 1983) have variously been proposed as sources of GA. The RNA-seq data indicated that the scutellum is the primary site of *de novo* GA synthesis, based on the high levels of transcripts for the major enzymes involved in the GA biosynthetic pathways, namely *GA13ox* (HORVU4Hr1G050930), *GA20ox* (HORVU5Hr1G124120), and *GA3ox* (HORVU3Hr1G022840) (Figs 4, 5; Supplementary Table S2). Transcripts encoding enzymes involved in GA synthesis are also found in the aleurone, albeit at lower abundance (Supplementary Table S2).

The third and fourth objectives were to identify the active form or forms of GA in the germinated barley grain, together with the biochemical pathways leading to these active forms. In earlier work, several chemical variants of GA were identified in germinated barley grain and isolated barley aleurone layers (Pharis and King, 1985). While exogenous GA_3_ was used in most experiments with isolated barley aleurone layers, the aleurone cells of oat, sorghum, maize, and certain varieties of barley are relatively insensitive to GA_3_ (Fincher, 1989).

The high abundance of transcripts of the *GA**13**ox* gene (HORVU4Hr1G050930), which encodes the enzyme that converts GA_12_ to GA_53_, and of transcripts for the *GA20ox* gene (HORVU5Hr1G124120), which encodes the enzyme that converts GA_53_ to GA_20_, shows that GA biosynthesis in the scutellum occurs via the 13-OH pathway (Fig. 4; Supplementary Table S2).

However, free bioactive GA_3_ and GA_4_ forms of the hormone, together with a GA_3_ conjugate, were also found in the aleurone (Supplementary Table S5). The presence of the GA_3_-G conjugate in the aleurone at 0 h indicates that at least some of it is deposited there during grain development, while its increase over the 96 h period also indicates that some *de novo* synthesis is occurring after germination. The free bioactive GA_4_ that is detectable throughout the 96 h period in the aleurone is synthesized via the non-13-OH pathway (Fig. 4), which is consistent with the presence of the *GA20ox* gene transcript (HORVU3Hr1G089980) in the aleurone (Supplementary Table S2). Some GA_4_ is also detected in the scutellum at 0 h, but not at the later time points (Supplementary Table S5). Yamada (1982) reported that GA_1_ was the predominant GA in germinated grain from two barley varieties and that GA_3_ was a minor GA; no GA_4_ was detected in that study. Our data indicate that both the 13-OH and non-13-OH pathways are used to synthesize several bioactive GAs in germinated barley, but it must be noted that active GA or related metabolites might also be synthesized via non-canonical pathways (Lenton and Appleford, 1991; Chandler, 2000; Hedden, 2019; Liu *et al.*, 2019).

The major product of GA synthesis in the scutellum is a GA_1_ glycosyl conjugate (GA_1_-G), some of which is synthesized in the developing grain and stored in the mature grain, but most of which is synthesized *de novo* in the scutellum (Supplementary Table S5). Glucosyl conjugates of various GAs have been implicated in the storage and long-distance transport of the hormones, in an inactive form (Schneider and Schliemann, 1994). Examination of the mass spectra of the GA_1_-G and GA_3_-G conjugates suggests that the glycosyl residue is almost certainly attached to the 3-OH of GA_1_ and GA_3_, where it would block the binding of GAs to the GID1 receptor (Shimada *et al.*, 2008; Supplementary Fig. S5) and hence block GA action. Release of the active GA would occur through the action of specific hydrolases that are presumably expressed only in the final target tissue and, in the case of the GA_1_-G conjugate, this occurs in the aleurone. There are a large number of genes encoding candidate glucosidases and esterases in barley and many are expressed in germinated grain (Supplementary Table S2A). However, the substrate specificities of most of these enzymes have not been rigorously assessed.

The fifth question relating to GA metabolism was to investigate whether turnover of active GA occurs in the germinated grain. Inactivation of active GAs and their immediate precursors is observed in both the 13-OH and non-13-OH pathways through the oxidation of carbon atom C(2) of the various GA forms (Fig. 4). Turnover of the active GAs and their immediate precursors was confirmed at the transcript level, where transcripts of *GA2ox* (HORVU3Hr1G029010) were moderately abundant in the aleurone but absent from the scutellum. However, transcripts of the *GA2ox* gene HORVU1Hr1G086810 were found at low levels in both the aleurone and the scutellum (Supplementary Table S2). Turnover was also confirmed at the assayed hormone level by the abundance of GA_8_, GA_34_, and GA_51_ degradation products in both the aleurone and the embryo (Fig. 4; Supplementary Table S5). The presence of GA_51_ indicated that the non-13-OH pathway can be aborted before the synthesis of active GA_4_.

The final question related to the possibility that the aleurone itself synthesizes, *de novo*, active GA during germination. This question impinges upon a potentially important signal transduction problem, namely whether bioactive, free GA forms, and/or inactive conjugates diffuse from the point of synthesis in the scutellum to the very distal tip of the grain, which in molecular and temporal terms is a very long distance. The major GA precursor metabolites, *ent*-kaurene and GA_53_, are present in the aleurone at 0 h and increase dramatically during germination (Supplementary Table S5). The much higher amounts of *ent-*kaurene in the embryo suggest that, although there is some of this precursor in the aleurone at 0 h, most of it arises by diffusion from the embryo from 6 h to 96 h. Similarly, some GA_53_ is present in the aleurone at 0 h and its levels increase up to 48 h (Supplementary Table S5). Thus, it is also likely that GA_53_ in the aleurone originates both by diffusion from the embryo and from synthesis in the aleurone during grain development.

When the hormone measurements are reconciled with the transcript data (Supplementary Tables S2, S5), it is clear that the biosynthesis of GA_1_, as its glycosyl conjugate GA_1_-G, occurs mainly in the scutellum via the 13-OH pathway. The smaller amounts of GA_3_ and its GA_3_-G glycoside conjugate are mostly synthesized *de novo* in germinated grain via the same pathway, but exclusively in the aleurone. The data also indicate that the low but relatively constant GA_4_ levels, which are found in the aleurone of germinated grain throughout the time course, are likely to be synthesized *de novo* under the influence of GA_1_, via the non-13-OH pathway (Fig. 4; Supplementary Table S5), despite the decreasing levels of the *GA20ox3* transcript (Fig. 5). The presence of GA_34_ in the aleurone throughout the time course further suggests that GA_4_ might be continuously synthesized and degraded after germination is initiated (Fig. 4; Supplementary Table S5). This is consistent with the early work of Atzorn and Weiler (1983), who showed that exogenous GA_1_ induced GA_4_ production in isolated barley aleurone layers and that the GA_4_ subsequently initiated α-amylase production. In contrast, Kaneko *et al.* (2003) concluded from their RT-PCR studies that the rice aleurone can perceive GAs but does not produce bioactive GAs, although levels of bioactive GAs, GA_53_, and GA conjugates were not reported in that study. Pearce *et al.* (2015) examined the phylogeny of the *GA3ox* gene family of grasses and showed that certain GA3ox enzymes convert GA_9_ to GA_131_, rather than to GA_4_. However, some of these apparent discrepancies might result simply from the different barley varieties used, given that the *GA20ox2* gene is knocked down in Green Revolution barley varieties and some compensation occurs in the transcription of other *GA20ox* genes (Pearce *et al.*, 2015; Xu *et al.*, 2017). Based on our RNA-seq and hormone measurements, we nevertheless conclude that GA_3_ and GA_4_ are synthesized in the aleurone of germinated barley and are prime candidates for ‘relaying’ active GA forms along the aleurone layer. However, the different conclusions reached by various groups working in this area suggest that further work is required to confirm a more general role for GA_4_ in relaying the hormonal signal along the aleurone layer in different varieties or cereal species.

**Fig. 7.**
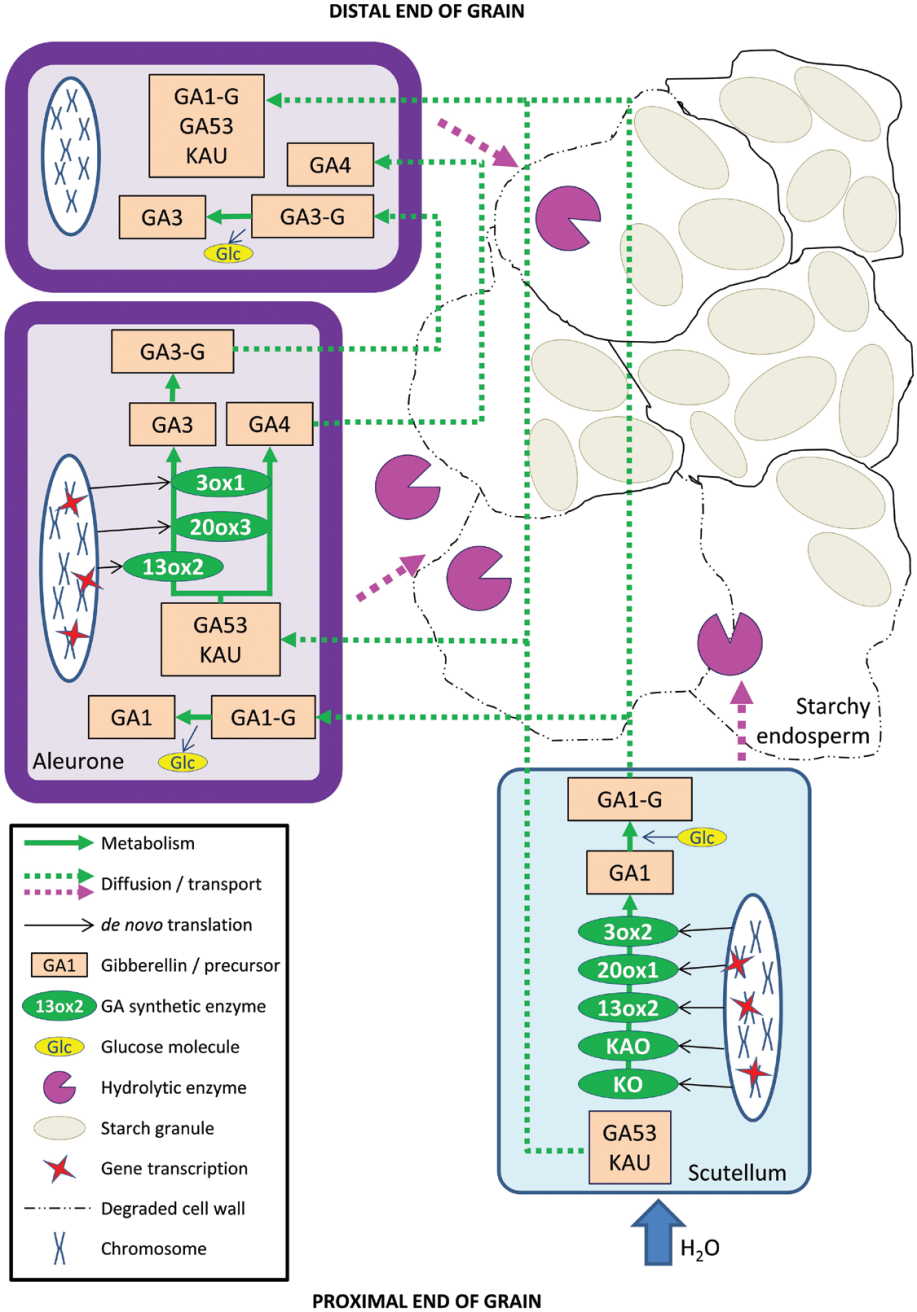
Proposed model of GA metabolism during barley grain germination. The uptake of water into the grain initiates active GA metabolism in the scutellar epithelial layer, where stored precursors *ent-*kaurene (KAU) and GA_53_ are converted to GA_1_, which is subsequently glycosylated to form GA_1_-G. The GA_1_-G, KAU, and GA_53_ are secreted and diffuse to the aleurone layer, where KAU and GA_53_ again act as precursor metabolites for active GA synthesis. The GA_1_-G is de-glycosylated and free GA_1_ causes transcription of *GA3ox*, *GA13ox*, and *GA20x* genes. The GA_3_ so produced may be glycosylated to form GA_3_-G before it is secreted, although it is also possible that free GA_3_ is secreted. The GA_3_-G, GA_3_, and GA_4_ from the aleurone layer diffuse to adjacent aleurone cells, where KAU and GA_53_, and some GA_1_-G have also diffused from the scutellum. The process continues from the proximal end towards the distal end of the aleurone layer; the concentration of the GA_1_-G is likely to become limiting towards the distal end of the grain, while GA_3_-G, GA_3_, and GA_4_ will be replenished en route. The long-distance transport of the hydrophobic *ent*-kaurene may involve lipid transport proteins. The red stars indicate gene transcription.

The RNA-seq data sets and measured concentrations of hormones and related metabolites not only provide a complete spatio-temporal picture of GA metabolism in the germinated barley grain at a level of detail not previously possible, but also provide answers to several longstanding anomalies in the germination literature. At the same time, the data demonstrate tight regulation of the pathways at multiple levels, which are depicted diagrammatically in the model shown in Fig. 7, although we are not yet able to rationalize the effects of mutations such as the GA-insensitive *slender* (*sln1*), in which GA and α-amylase synthesis are somehow uncoupled (Chandler *et al.*, 2002). We also acknowledge that the measured values of the hormones and related metabolites are semi-quantitative in nature, but the standards used during chromatography, coupled with the mass spectral data, enable the identification of metabolites actually present in the different tissues, and the time courses allow the trends in their concentrations to be monitored.

In summary, our data indicate that the GA precursors *ent*-kaurene, *ent*-kaurenoic acid, and GA_53_ are found in the scutellum of mature, ungerminated grain and are converted predominantly to the GA_1_ glycosyl conjugate after the initiation of germination. This GA_1_-G probably diffuses from the scutellum to the aleurone during germination, where it triggers the production of other GAs, including GA_3_, GA_3_-G, and GA_4_. Despite the possibility that this sequence of events and the metabolites involved vary between barley varieties, it appears that in cv. Navigator these forms stimulate neighbouring aleurone cells, and in this way the concentrations of the active forms are maintained and ensure their signal is transmitted to the distal end of the grain (Fig. 7). The processes modelled in Fig. 7 have provided the plant, through evolution, with multiple back-up options designed to maximize the chances of successful germination and hence survival into the next generation. The identification of important individual genes from multigene families provides targets for increasing crop productivity via conventional or mutagenesis breeding. In addition, the complete RNA-seq data sets provided here can be further mined to define genes that mediate other important biological processes in germinated grain.

## Supplementary data

Supplementary data are available at *JXB* online.

Fig. S1. Multidimensional scaling analysis of transcription patterns in five germinated grain tissues.

Fig. S2. Numbers of differentially expressed genes (DEGs).

Fig. S3. Overlaps in differentially expressed genes (DEGs) between tissues and across the 96 h time course of the aleurone al1 tissue.

Fig. S4. Mass spectra of standard GA_1_ and GA_3_, together with spectra for the corresponding GA_1_-G and GA_3_-G glycosides.

Table S1. LC-MS/MS parameters used for the manual identification of the detected gibberellins

Table S2. Complete data set of transcript abundance and log_2_(fold change) of genes in the aleurone, scutellum, and embryo of germinated barley grain.

Table S3. Transcript abundance of genes mentioned in the text of this manuscript.

Table S4. Genes involved in GA metabolism. Two genes, *KO1* and *GA2ox5*, are each represented by two HORVU in the new genome annotation. The table does not include one locus, HORVU2Hr1G036450, a likely pseudogene that encodes a small section homologous to KSL proteins.Table S5. Levels of hormones and related metabolites in the embryo and aleurone of germinated barley grain.

erz546_suppl_Supplementary_Table_S1

erz546_suppl_Supplementary_Tables_S2-S4

erz546_suppl_Supplementary_Figures_S1-S4

erz546_suppl_Supplementary_Data

## Data Availability

RNA-seq data have been deposited in the NCBI SRA database under BioProject ID PRJNA53351.
