# Supplementary material for: Transcriptional and biochemical analyses of gibberellin expression and content in germinated barley grain
Source: J Exp Bot. 2019 Dec 10;71(6):1870–84. doi: 10.1093/jxb/erz546 (PMC7242073; doi:10.1093/jxb/erz546)
Supplement: erz546_suppl_Supplementary_Table_S1 [file erz546_suppl_supplementary_table_s1.pdf]

Supplementary Table S1 LC-MS/MS parameters used for the manual identification of the detected gibberellins.

Molecule	Mode	Formula	Adduct	m/z	Retention time (min)	Characteristic ions (m/z transitions)*	Present in barley (MS2)	Standard
**GA hormone**								
GA_1	Negative	$C_{19}H_{24}O_6$	-[H^+]	347.1495	21.2–21.5	**259**, 273, 303	✓	✓
GA_3	Negative	$C_{19}H_{22}O_6$	-[H^+]	345.1338	21.2–21.9	143, 221, **239**, 301		✓
			-[H_2O]-[H^+]	327.1233			low	
GA_4	Negative	$C_{19}H_{24}O_5$	-[H^+]	331.1545	40.2–41.9	213, 257, 269, **287**, 313		✓
			+[$COOH^-$]-[H_2O]	359.1495			✓	
GA_7	Negative				39.8	168, 211, **223**, 287		✓
GA_8	Negative	$C_{19}H_{24}O_7$	-[H^+]	363.1444	18.6–19.1	119, 257, **275**, 321	✓	
GA_{19}	Negative	$C_{20}H_{26}O_6$	-[H^+]	361.1651	45.8–46.2	133, 203, 229, 255, **273**, 317	✓	
GA_{20}	Negative	$C_{19}H_{24}O_5$	-[H^+]	331.1545	31.2	147, 173, 219, 225, 250, **287**		✓
GA_{34}	Negative	$C_{19}H_{24}O_6$	-[H^+]	347.1495	36.1–36.7	199, 241, **259**, 303	✓	
GA_{44}	Negative	$C_{20}H_{26}O_5$						
GA_{51}	Negative	$C_{19}H_{24}O_5$	-[H^+]	331.1545	36.3–38.0	182, 219, 243, **287**	✓	✓
GA_{53}	Negative	$C_{20}H_{28}O_5$	-[H^+]	347.1858	41.6–42.5	189, 233, 303, **329**	✓	✓
**GA-glycoside**								
GA_1-G	Negative	$C_{25}H_{34}O_{11}$	-[H^+]	509.2023	33.4–33.9		✓	
GA_3-G	Negative	$C_{25}H_{31}O_{11}$	-[H^+]	507.1866	30.1–30.9		✓	
**GA precursor**								
ent-kaurene	Positive	$C_{20}H_{32}$	+[H^+]	273.2582	50.9–52.2		✓	
ent-kaurenoic acid	Negative	$C_{20}H_{30}O_2$	-[H^+]	301.2168	69.1–69.5		✓	✓

* m/z transitions from Chiwocha *et al.* (2003), Delatorre *et al.* (2017) and Urbanová *et al.*, (2013). Major signals (Urbanova *et al.*, 2013) are indicated in bold; signals not detected in this analysis are indicated in **blue**. m/z transitions are labelled in Supplementary Fig S4. Note that retention times of the glycosides are larger than GA_1 and GA_3 and that this is not what might be expected on a C18 column. However, elution times on C18 columns can be affected by more than just polarity. Intermolecular reactions during chromatography, interactions with the column matrix and compound conformation can all influence the retention time. It is relevant here that a β-glucosyl residue has a highly hydrophobic face (involving the axial C-H groups on C1, C3 and C5). If this face were exposed to the C18 chain, then it would most probably slow down the elution of the conjugate. One could also speculate that the equatorial OH groups on C2, C3, C4 and C6 could fold back and form hydrogen bonds with the COOH and OH groups of the GA, which might also decrease the polarity of the exposed surface of the molecule.
