# Supplementary material for: Transcriptional and biochemical analyses of gibberellin expression and content in germinated barley grain
Source: J Exp Bot. 2019 Dec 10;71(6):1870–84. doi: 10.1093/jxb/erz546 (PMC7242073; doi:10.1093/jxb/erz546)
Supplement: erz546_suppl_Supplementary_Figures_S1-S4 [file erz546_suppl_supplementary_figures_s1-s4.pdf]

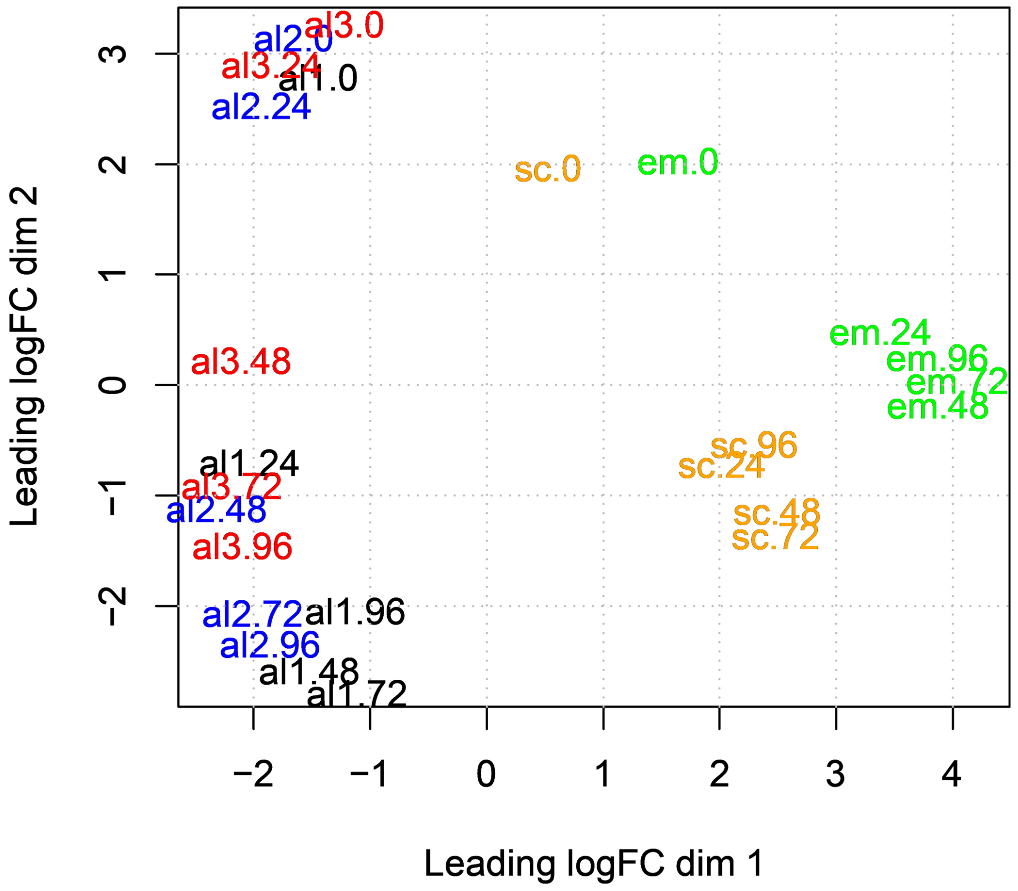

Supplemenatory Figure S1: Multi-dimensional scaling analysis of transcription patterns in five germinated grain tissues.
Shown is a principle component analysis for the five tissues and five time points analysed based on the transcript abundances (transcripts per million, TPM) determined by the RNA-seq experiment. Abbreviations: al1, al2, al3, aleurone proximal, central,distal sections, respectively; em, embryo; sc, scutellum.

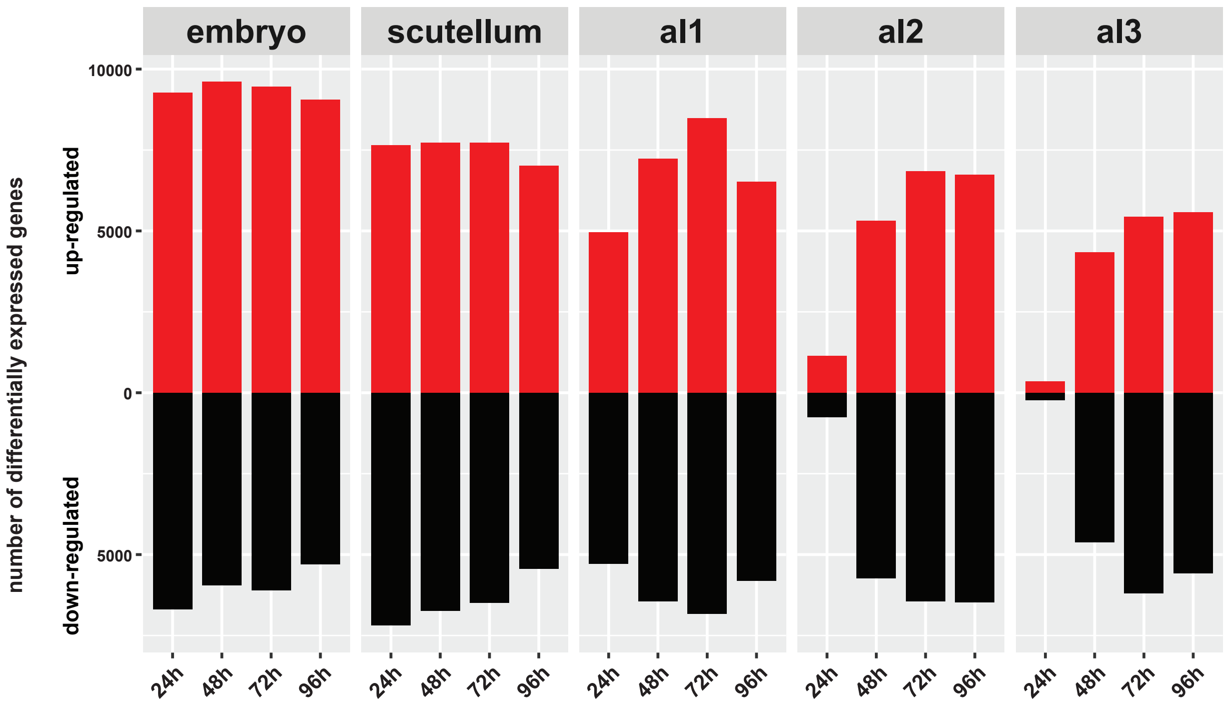

Supplementary Figure S2: Numbers of differentially expressed genes (DEGs).
The bar chart shows the number of differentially up- or downregulated genes in the five analysed tissues relative to the 0 h time point. Abbreviations: al1, al2, al3, aleurone proximal, central,distal sections, respectively.

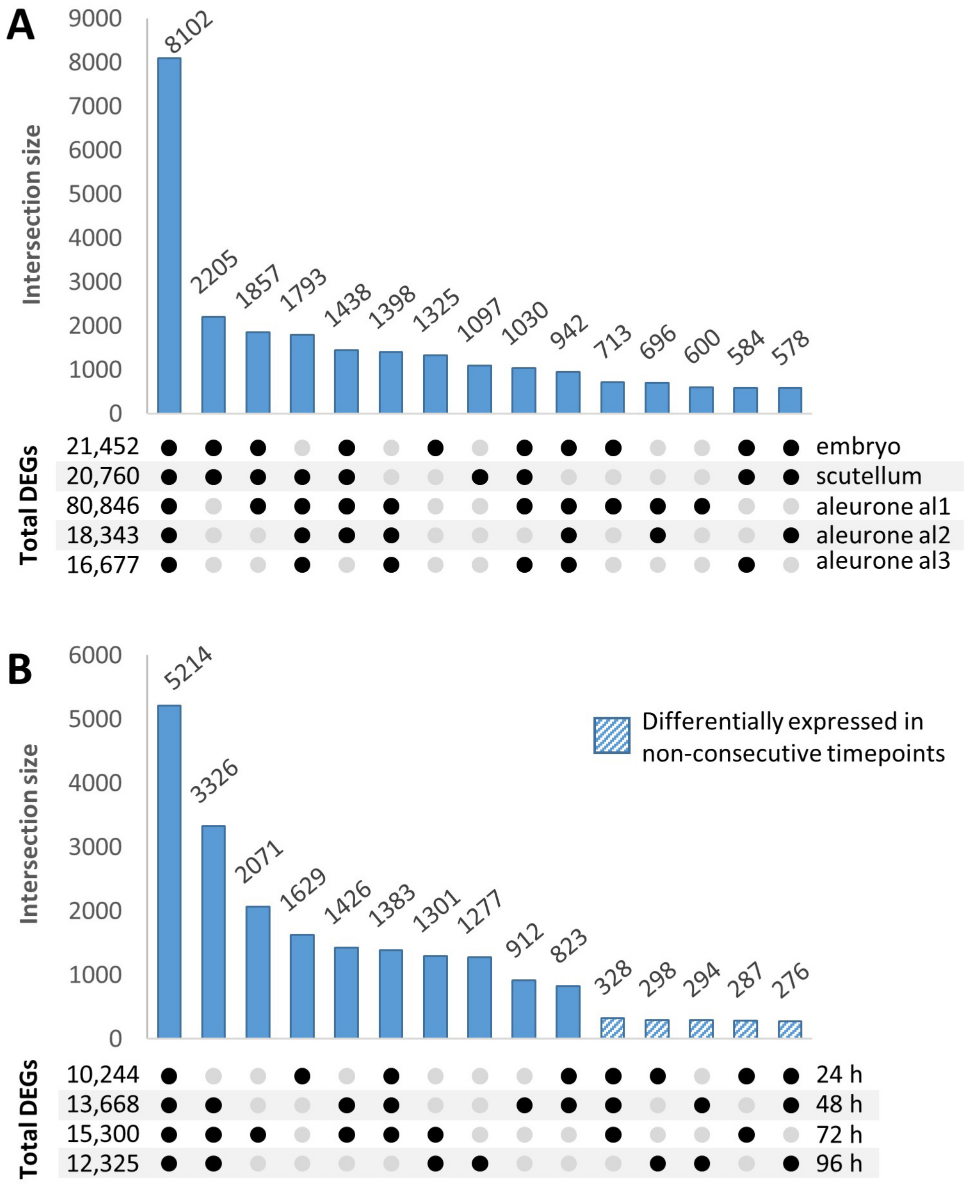

Figure S3. Overlaps in differentially expressed genes (DEGs) between tissues (A) and across the 96 h time course (B) of the aleurone al1 tissue.

Supplementary Figure S4 Mass spectra of standard GA_1 and GA_3, together with spectra for the corresponding GA_1-G and GA_3-G glycosides. Mass ions common to the GA and its corresponding GA-glycoside are indicated with green asterisks. Ions characteristic of established m/z transitions for each hormone are labelled (Chiwocha *et al.*, 2003; Urbanová *et al.*, 2013; Delatorre *et al.*, 2017). MRM denotes multiple reaction monitoring.

GA_1 standard

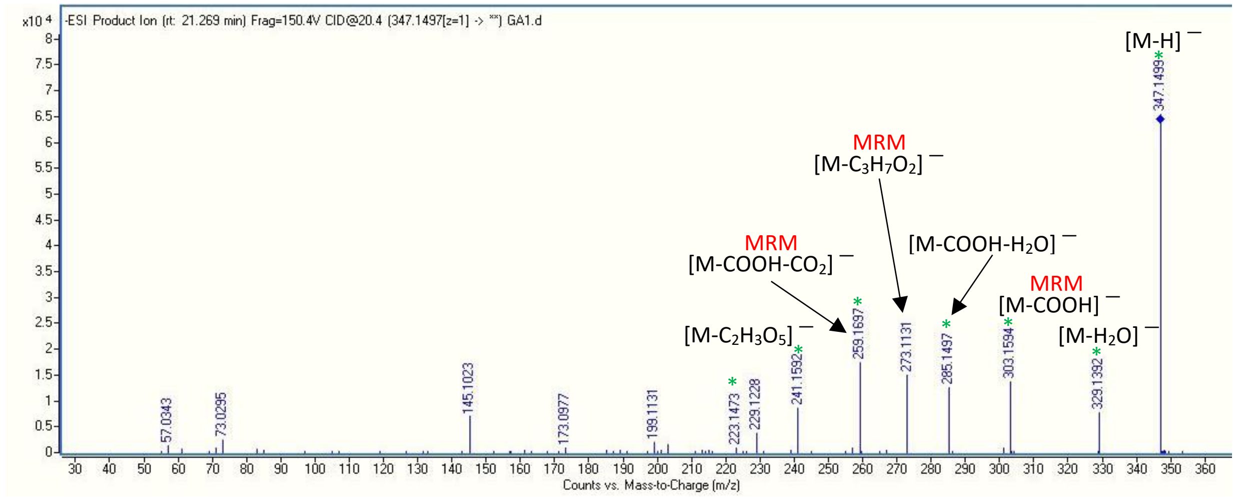

GA_1-G (Δ=162)

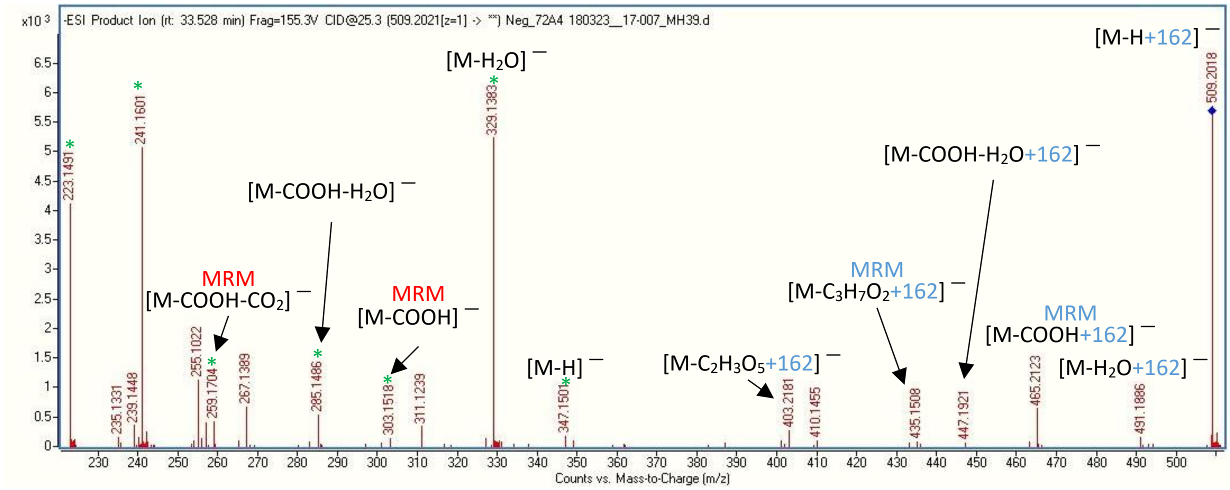

* Common peaks in both spectra

GA_3 standard

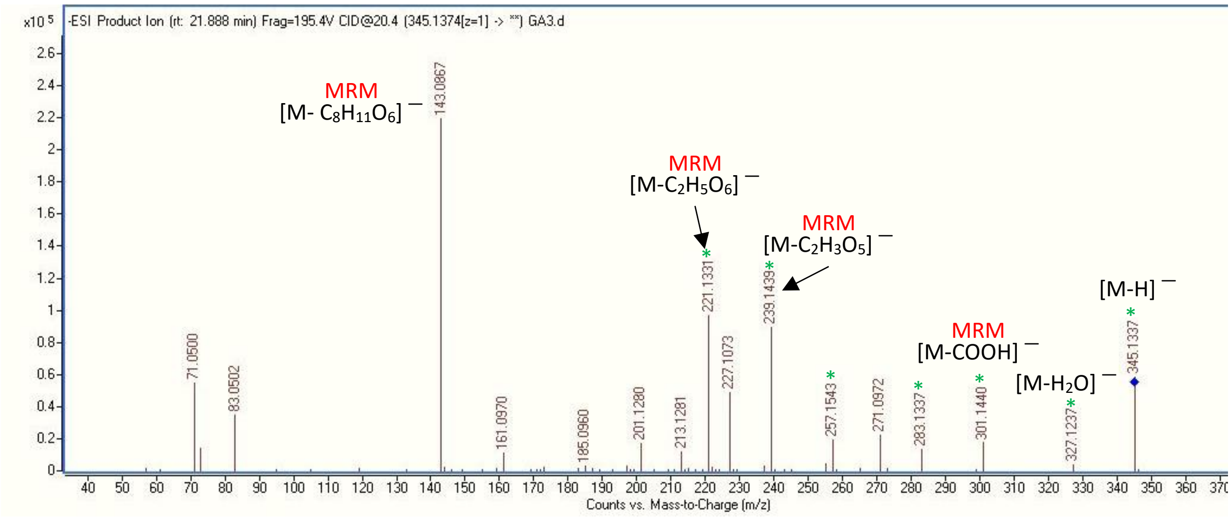

GA_3-G (Δ=162)

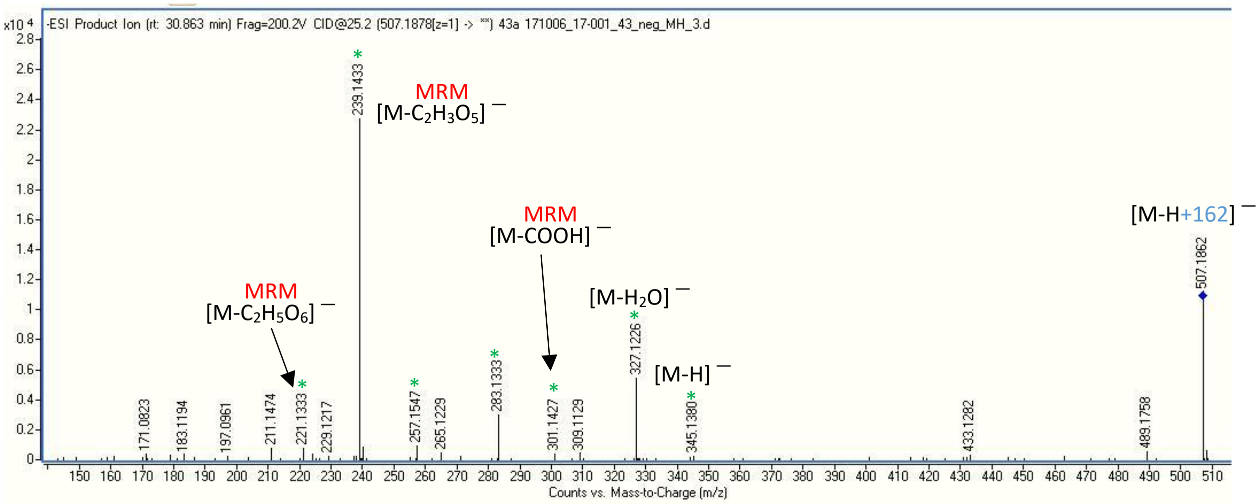

Footnote: The molecular ions ($M-H^-$) of the glycosides correspond to the molecular ions of GA_1 and GA_3 plus 162 (509 and 507). The 162 mass unit is the precise size of the anhydo-hexose that is linked to the individual GAs. The hexose unit might be glucose, galactose or mannose, but we conclude that the weight of evidence in the literature strongly suggests it will be glucose. Further, typical GA and glycosyl fragmentation patterns are observed in both the glycosylated and unglycosylated forms.

The presence of the m/z ions of 239 and 241 for GA_3 and GA_1, respectively, is predicted to correspond to the molecular ion of the GAs, minus $C_2H_3O_5$ (m/z 107). Because these m/z 239 and m/z 241 ions are found in both the glycosylated and non-glycosylated spectra, it can be concluded that these fragments do not contain a glycosyl residue. Furthermore, the MRM ions of $[M-C_2H_3O_5]^-$ in both the GA and glycoside spectra is consistent with the loss of two COOH groups and one OH group. Given that both GA_1 and GA_3 contain only six O atoms (Figure 4), it might be argued that removal of five O atoms in these m/z 239 and 241 ions suggests the glycosyl residues are attached in glycosidic linkage to the single remaining O atom of a OH group rather than as an ester linkage to a COOH group.

The consistent difference of two mass units in many of the GA-conjugate fragments indicates that these fragments are from the end of the molecule that contains the double bond that differentiates GA_1 from GA_3 (Figure 4). Hence, it might be argued that the glycosyl residue is conjugated to the 3-OH, rather than the 13-OH. However, caution must be taken in coming to this conclusion, which awaits confirmation through the analyses of standard GA_1-G and GA_3-G; neither are currently available.
