# Supplementary material for: Transcriptional and biochemical analyses of gibberellin expression and content in germinated barley grain
Source: J Exp Bot. 2019 Dec 10;71(6):1870–84. doi: 10.1093/jxb/erz546 (PMC7242073; doi:10.1093/jxb/erz546)
Supplement: erz546_suppl_Supplementary_Data [file erz546_suppl_supplementary_data.docx]

**Supplementary data**

Supplementary data are available at *JXB* online.

Fig. S1. Multi-dimensional scaling analysis of transcription patterns in five germinated grain tissues.

Fig. S2. Numbers of differentially expressed genes (DEGs).

Fig. S3. Overlaps in differentially expressed genes (DEGs) between tissues (A) and across the 96 h time course (B) of the aleurone al1 tissue.

Fig. S4. Mass spectra of standard GA_1_ and GA_3_, together with spectra for the corresponding GA_1_-G and GA_3_-G glycosides.

Table S1 LC-MS/MS parameters used for the manual identification of the detected gibberellins

Table S2. Complete dataset of A) transcript abundance and B) log_2_(fold change) of genes in the aleurone, scutellum and embryo of germinated barley grain.

Table S3. Transcript abundance of genes mentioned in the text of this manuscript.

Table S4. Levels of hormones and related metabolites in the embryo and aleurone of germinated barley grain.
